# A neural network solves, explains, and generates university math problems by program synthesis and few-shot learning at human level

**DOI:** 10.1073/pnas.2123433119

**Published:** 2022-08-02

**Authors:** Iddo Drori, Sarah Zhang, Reece Shuttleworth, Leonard Tang, Albert Lu, Elizabeth Ke, Kevin Liu, Linda Chen, Sunny Tran, Newman Cheng, Roman Wang, Nikhil Singh, Taylor L. Patti, Jayson Lynch, Avi Shporer, Nakul Verma, Eugene Wu, Gilbert Strang

**Affiliations:** ^a^Department of Electrical Engineering and Computer Science, Massachusetts Institute of Technology, Cambridge, MA 02139, United States of America;; ^b^Department of Computer Science, Columbia University, New York, NY 10027, United States of America;; ^c^Department of Mathematics, Massachusetts Institute of Technology, Cambridge, MA 02139, United States of America;; ^d^Department of Mathematics, Harvard University, Cambridge, MA 02138, United States of America;; ^e^Media Lab, Massachusetts Institute of Technology, Cambridge, MA 02139, United States of America;; ^f^Department of Physics, Harvard University, Cambridge, MA 02138, United States of America;; ^g^School of Computer Science, University of Waterloo, Waterloo, ON N2L 3G1, Canada;; ^h^Department of Physics and Kavli Institute for Astrophysics and Space Research, Massachusetts Institute of Technology, Cambridge, MA 02139, United States of America

**Keywords:** neural networks, mathematics courses, answering, explaining, and generating questions

## Abstract

We demonstrate that a neural network pretrained on text and fine-tuned on code solves mathematics course problems, explains solutions, and generates questions at a human level. We automatically synthesize programs using few-shot learning and OpenAI’s Codex transformer and execute them to solve course problems at 81% automatic accuracy. We curate a dataset of questions from Massachusetts Institute of Technology (MIT)’s largest mathematics courses (Single Variable and Multivariable Calculus, Differential Equations, Introduction to Probability and Statistics, Linear Algebra, and Mathematics for Computer Science) and Columbia University’s Computational Linear Algebra. We solve questions from a MATH dataset (on Prealgebra, Algebra, Counting and Probability, Intermediate Algebra, Number Theory, and Precalculus), the latest benchmark of advanced mathematics problems designed to assess mathematical reasoning. We randomly sample questions and generate solutions with multiple modalities, including numbers, equations, and plots. The latest GPT-3 language model pretrained on text automatically solves only 18.8% of these university questions using zero-shot learning and 30.8% using few-shot learning and the most recent chain of thought prompting. In contrast, program synthesis with few-shot learning using Codex fine-tuned on code generates programs that automatically solve 81% of these questions. Our approach improves the previous state-of-the-art automatic solution accuracy on the benchmark topics from 8.8 to 81.1%. We perform a survey to evaluate the quality and difficulty of generated questions. This work automatically solves university-level mathematics course questions at a human level and explains and generates university-level mathematics course questions at scale, a milestone for higher education.

## Significance

We demonstrate that a neural network automatically solves, explains, and generates university-level problems from the largest Massachusetts Institute of Technology (MIT) mathematics courses at a human level. Our methods combine three innovations: 1) using recent neural networks pretrained on text and fine-tuned on code rather than pretrained on text; 2) few-shot learning synthesizing programs that correctly solve course problems automatically; and 3) a pipeline to solve questions, explain solutions, and generate new questions indistinguishable by students from course questions. Our work solves university-level mathematics courses and improves upon state-of-the-art, increasing automatic accuracy on randomly sampled questions on a benchmark by order of magnitude. Implications for higher education include roles of artificial intelligence (AI) in automated course evaluation and content generation.

Until this work, it was widely believed that neural networks could not solve advanced mathematics problems (1). However, the previous unsuccessful studies used only text-based pretraining. We now demonstrate that a neural network, OpenAI Codex, that is pretrainedon text and fine-tuned on code automatically answers 81% of university-level mathematics problems by program synthesis using few-shot learning.

**Fig. 1.**
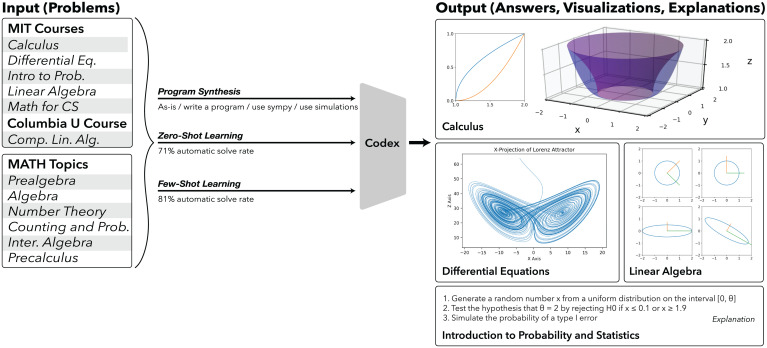
We apply a neural network, OpenAI Codex, to solve, explain, and generate mathematics problems. We randomly sample the input math problems from MIT and Columbia University courses and the MATH dataset (*Left*). We use zero-shot and few-shot learning to automatically generate programs that solve 81% of the questions. We then use Codex to explain the generated programs. The generated programs can output diverse forms of answers, like printing a numerical answer or generating a plot (*Right*): for example, in Calculus, the volume generated by rotating the finite two-dimensional region bounded by two two-dimensional graphs about the plotted axis (*Top Right*); in Differential Equations, the Lorenz strange attractor (*Middle Right*); in Linear Algebra, the geometry of the SVD (*Middle Right*). An example of Codex’s ability to produce line-by-line explanations of synthesized programs is demonstrated for a problem from Introduction to Probability and Statistics (*Bottom Right*).

Fig. 1 illustrates several example problems: computing the volume generated by rotating the graph of a single variable function around an axis, computing the Lorenz attractor and its projection, and computing and demonstrating the geometry of a singular-value decomposition (SVD). We show that a single machine-learning model can solve these example problems and solve a wide variety of mathematics courses at scale.

## Related Work.

Transformers are deep-learning architectures based only on attention mechanisms (2) that do not use recurrent neural networks or convolutional neural networks. Transformer-based language models have enjoyed tremendous success across various natural language-processing (NLP) tasks, including zero-shot and few-shot language tasks (3). However, these models have largely failed to solve math problems (4–6). In particular, previous work using transformers, such as GPT-3 (3), has failed to solve mathematics problems because the transformers were pretrained on text alone. Using few-shot learning and chain-of-thought (CoT) prompting (7) improves the mathematical reasoning ability of GPT-3; however, without code, GPT-3 with few-shot learning and CoT still fails on university-level mathematics problems and the MATH benchmark.

Pretraining a transformer is computationally expensive and often involves vast amounts of unlabeled data. The most common optimization objectives for pretraining language models are 1) masked word prediction, predicting a random deleted word in a sentence or predicting the next word, and 2) classifying whether two sentences follow each other. This computationally expensive step is usually done once, followed by a relatively fast fine-tuning step. In fine-tuning, the pretrained model is tuned using a specific dataset or task.

This work demonstrates that OpenAI’s Codex (8), a transformer that has been pretrained on text and then fine-tuned on code, generates programs (i.e., conducts program synthesis) that solve math problems at scale and, with few-shot learning, automatically solves 81% of the math course problems.

Previous work has seen modest success on simpler or specialized mathematics problem benchmarks. Techniques based on cotraining output to verify (9, 10) or predict expression trees (11–16), such as MAWPS and Math23k, are able to solve elementary school-level math problems with over 81% accuracy. However, these approaches do not extend to high-school, math Olympiad, or university-level courses. Cotraining paired with graph neural networks (GNNs) to predict arithmetic expression trees is able to solve university-level problems in machine learning (17) with up to 95% accuracy. However, that work is limited to numeric answers and overfits a specific course, which does not generalize to other courses.

## Major Contributions.

**Fig. 2.**
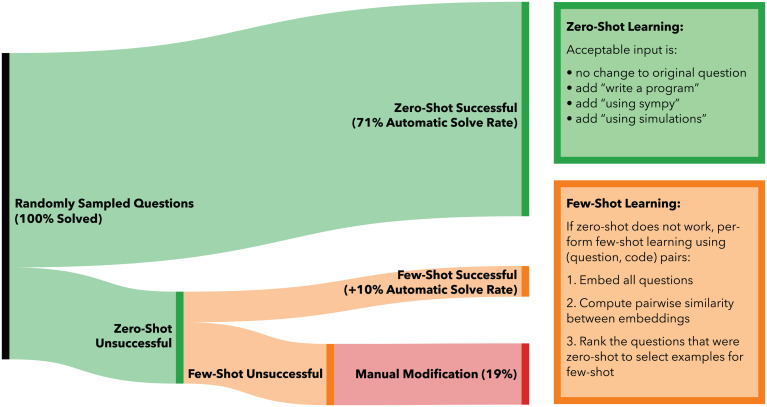
We select a random sample of questions from each course or topic that do not contain input images or require proofs. A language model pretrained on text (GPT-3 text-davinci-002) automatically solves only 18% (for courses) and 25.5% (for the MATH benchmark topics) of these questions. In contrast, using zero-shot learning with a network pretrained on text and fine-tuned on code (OpenAI Codex code-davinci-002), we synthesize programs that automatically solve 71% (for courses) and 72.2% (for the MATH benchmark topics) of the questions. Using the same network but using few-shot learning, we automatically solve 81% (for courses) and 81.1% (for the MATH benchmark topics) of the questions. We use the nearest embedded zero-shot questions and their synthesized code for few-shot learning. The remaining 19% of the course questions and 18.9% of MATH benchmark topic questions are manually prompted to solve the question.

Our main contribution, as shown in Fig. 2, is demonstrating that a single neural network model, OpenAI Codex, automatically solves 81% of randomly selected university-level mathematics problems (from six Massachusetts Institute of Technology [MIT] mathematics courses and one Columbia University course) by using program synthesis and few-shot learning. We also automatically explain the solutions and generate new questions, a process requiring only seconds per problem. The courses are listed in Table 1. We randomly sample 25 questions per course, and the problems are solved as is or with minor contextual information that is automatically applied. The neural network outputs an executable program that answers the problem when prompted with the question. Furthermore, our method explains the solutions and generates new problems nearly indistinguishable from human-written problems.

**Table 1.**
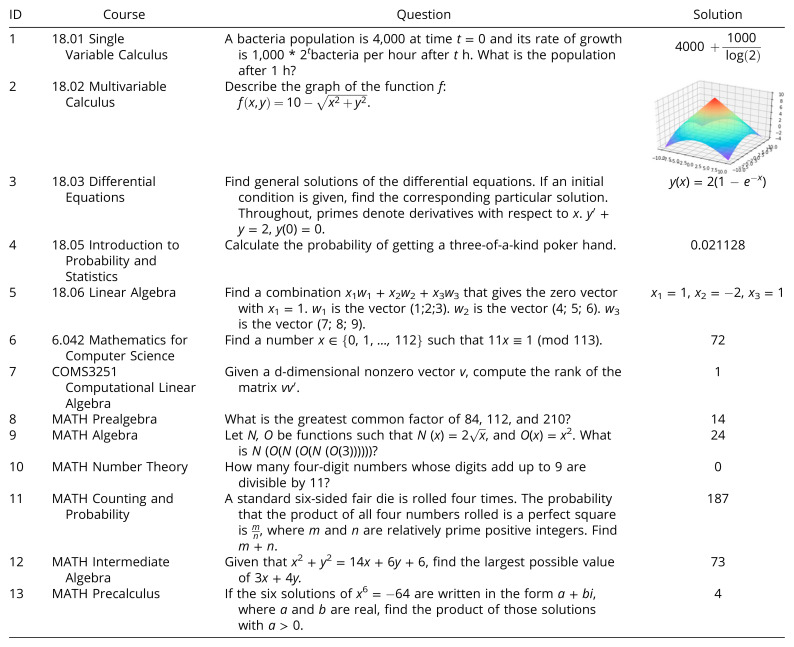
Example questions and solutions from six MIT courses (18.01, 18.02, 18.03, 18.05, 18.06, 6.042), one Columbia University course (COMS3251), and six topics from the MATH dataset. The solutions can contain numerical answers, equations, plots, or other modalities

This methodology increases the solution accuracy on the MATH benchmark (5) from 8.8% accuracy using previous state-of-the-art methods to 81.1% accuracy using automatic few-shot learning. The MATH benchmark measures the mathematical problem-solving ability of neural network models with challenging problems sourced from high-school math competitions, such as the AMC 10,* AMC 12, and AIME.^†^

The methods we propose are simple and broadly applicable. The first one uses a transformer model pretrained on text and fine-tuned on code so that it is adept at synthesizing programmatic solutions. The second one uses zero-shot learning of the questions as is or automatically added contextual information about the problem or program. The third one uses few-shot learning based on question–code pairs of similar questions that have been solved, found by using the cosine similarity of the question embeddings.

## Methods

### Dataset.

We randomly sample 25 questions from each of the seven courses: MIT’s 18.01 Single Variable Calculus, 18.02 Multivariable Calculus, 18.03 Differential Equations, 18.05 Introduction to Probability and Statistics, 18.06 Linear Algebra, and 6.042 Mathematics for Computer Science and Columbia University’s COMS3251 Computational Linear Algebra. For the MATH dataset, we randomly sample 15 questions from six topics in the dataset (Algebra, Counting & Probability, Intermediate Algebra, Number Theory, Prealgebra, and Precalculus). We validate that our results are not merely overfitting training data by solving questions from a new Computational Linear Algebra course COMS3251 that is unavailable online and was unseen by Codex when trained. We automatically obtain correct answers for 81% of the randomly sampled university math course questions and 81.1% of the MATH benchmark questions. Before this work, the previous state of the art on this benchmark was 8.8% (4).

### Workflow.

**Fig. 3.**
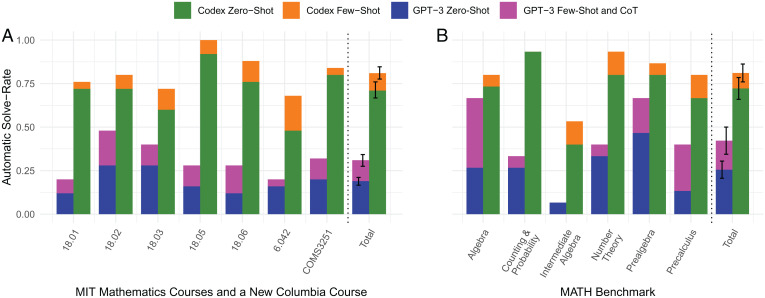
Comparison of the automatic solve rates on (*A*) MIT math courses and a Columbia University course and on (*B*) MATH benchmark dataset. The latest OpenAI GPT-3 (text-davinci-002), a transformer pretrained on text, achieves on the MIT math courses (*A*) 18.8% with zero-shot and 30.8% with few-shot and CoT and on the MATH benchmark (*B*) 25.5% with zero-shot and 42.2% with few-shot and CoT. In contrast, program synthesis using the latest OpenAI Codex (code-davinci-002), a transformer pretrained on text and fine-tuned on code, achieves automatic solve rates on the MIT math courses of (*A*) 71.1% with zero-shot learning and 81.1% with few-shot learning and on the MATH benchmark (*B*) 72.2% with zero-shot learning and 81.1% with few-shot learning.

Our method takes a course problem as input and synthesizes a program that, when run, outputs the solution. Fig. 3 compares the percentage of automatically solved questions for each course using our zero-shot learning and few-shot learning approaches with the latest GPT-3 (text-davinci-002) and Codex (code-davinci-002) versions. The error bars on the totals are SEs.

**Fig. 4.**
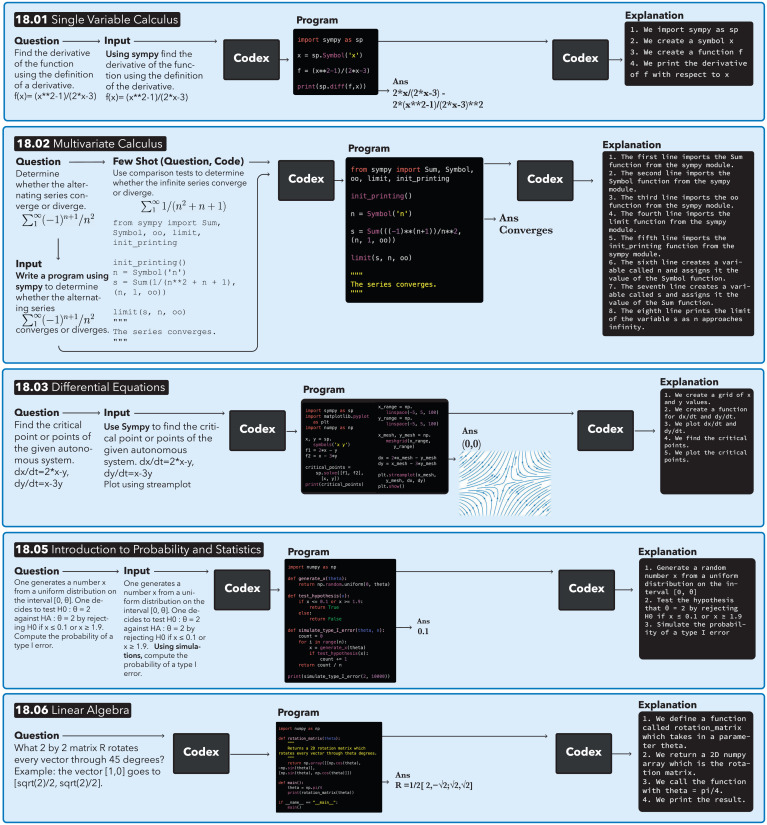
Example pipelines automatically solve questions from MIT mathematics courses and explain the solutions. In the 18.01 Single Variable Calculus Zero-Shot example, given a question and the automatically generated prefix “using SymPy,” Codex is prompted and outputs a program. Running the program results in equations that are the correct answer. The program is then fed to Codex again with an automatic prompt, resulting in a generated code explanation. In the 18.02 Multivariable Calculus Few-Shot example, given a question, the prefix “write a program using SymPy” is automatically generated. The question is embedded with the other zero-shot questions in the course. The nearest zero-shot question and its corresponding code are used as a few-shot example. The few-shot example pair and the input question are fed into Codex, which generates a program that solves the question. The question, program, and prompt for explanation are fed into Codex to generate the explanation. In the 18.03 Differential Equations Zero-Shot example, the answer is both a vector and a plot. In the 18.05 Introduction to Probability and Statistics Zero-Shot example, given the question, a probabilistic program is generated by adding “using simulation” to the prompt. In the 18.06 Linear Algebra Zero-Shot example, the output answer is the correct vector.

Fig. 4 shows examples of automatic workflows for solving course questions and generating explanations using Codex. The panels show the original question, the automatic augmentation with context, the resulting synthesized program, the executed output answer that is the solution, and the explanation of the solution program. Questions are given to Codex either as is or by automatically adding minor context, as described below. The output answer may be of numerous modalities. In the examples featured in Fig. 4, the output answers are an equation (18.01), a Boolean value (18.02), a plot (18.03), a numerical value (18.05), and a vector (18.03 and 18.06).

### Automatic Contextualization.

#### Programming language context.

Best results are obtained when the Codex prompt specifies that a program should be written and specifies which programming language should be used. We add the text “write a program” before the question and focus on the Python programming language by placing the text within Pythonic triple quotes like a docstring.

#### Library context.

Likewise, the best results are obtained when the Codex prompt specifies which programming package should be used. For instance, we may add the Python library SymPy as context (Fig. 4, *Top*, 18.01), specifying that the program synthesized to solve the problem should use this package.

**Fig. 5.**
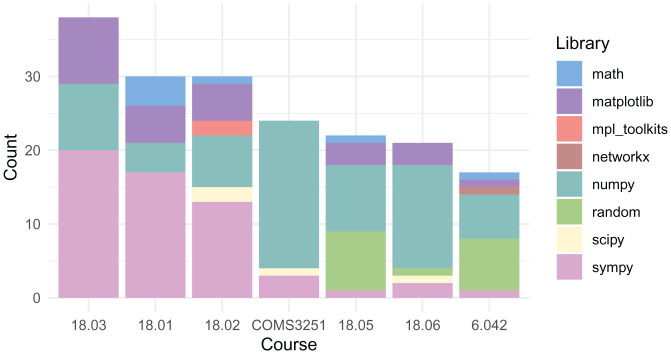
Imported Python programming libraries by course: NumPy is used by nearly all courses. Matplotlib is used in courses with questions that involve plotting. Sympy is used by most of the courses and SciPy by half of the courses.

Fig. 5 shows the Python programming packages used by each course. Each colored stacked bar represents the number of questions in the class using that package. All courses use NumPy and Sympy. Matplotlib is used in classes with questions that require plotting. Around half of the courses use math, random, and SciPy. The usage patterns of these courses are incorporated automatically in our approach, as we specify only SymPy or plot-related imports; these other package imports are automatically synthesized.

### Automatic Zero-Shot and Few-Shot Learning.

Zero-shot learning synthesizes a program from the original question or the automatically augmented question without examples. This method automatically solves 71% of the questions. Next, we describe the few-shot learning process in detail. If the question is not solved, we do the following: we embed all the questions using OpenAI’s text-similarity-babbage-001 embedding engine, which embeds the questions onto a 2,048-dimensional space. Then, we calculate the most similar solved questions to the unsolved question from within its course using the cosine similarity of the embeddings. We take the most similar question and its corresponding code and use these as few-shot examples for the new question. If the generated code does not output the correct answer, we add another solved question–code pair, using the next similar solved question each time. We found that using up to five examples for few-shot learning works well in practice, increasing the total number of questions automatically solved from 71% using zero-shot learning to 81% using few-shot learning. Fig. 4 (18.02) demonstrates few-shot learning.

#### Simulation.

Fig. 4 (18.05) shows an example from Probability and Statistics where the question is turned into a probabilistic programming task that generates simulations to compute an empirical statistic.

### Manual Prompt Modification.

#### Question tidying.

While 81% of the questions are automatically solved by zero-shot and few-shot learning, 19% of the questions may require manual editing to be solved by Codex. These questions may be vague or contain redundant information (e.g., reference movie characters or current events) and require tidying to extract the essence of the question. Question tidying primarily involves removing redundant information, breaking down long sentence structures into smaller components, and converting prompts into a programming format.

#### Interaction for visualization.

Another form of manual prompting occurs when an answer involves a plot and requires multiple steps to generate a visually pleasing and clear plot. These special cases, which are among the remaining 19% of the questions, require interactively prompting Codex until reaching the desired visualizations.

### Automatic Explanation.

Explanations are generated automatically using the question, the code generated by Codex when prompted with the question, and a prompt consisting of three quotes followed by the text “Here is what the above code is doing: 1.” This prompt is given after both the question and the generated code since the code may be a lossy representation of the question. The result is a step-by-step explanation of the solution code given to Codex.

### Generation of Questions and Their Human Evaluation.

We also use Codex to generate new questions for each course. This is done by creating a numbered list of human-written questions from each class. This list is cut off after a random number of questions, and the result is used to prompt Codex to generate the next question. This process is repeated to create many new questions for each course.

To evaluate the generated questions, we survey MIT students who have taken these courses or their equivalents to compare the quality and difficulty of machine-generated questions with human-written questions for each of the courses. The Institutional Review Board (IRB) that approved the survey is MIT IRB Exempt Id E-3792. The survey was optional and included informed consent, with the following description: “We are conducting a survey to assess the quality and difficulty of automatically generated questions for science, technology, engineering, and mathematics (STEM) courses. You will be presented with a series of blocks consisting of questions, either human-written (taken from an actual course) or generated with machine learning, but you will not be told the source of a given question. For each question, you will be asked (a) whether you think the question is human-written or machine-generated, (b) whether the question is appropriate for the given course, and finally, (c) how you would rate the difficulty of the question. Please carefully read each question and answer to the best of your ability.” We randomly sampled five original, human-written questions and five generated questions for each of the six MIT courses. Students are asked to read these 10 questions per course in the survey, mixed and presented randomly.

**Fig. 6.**
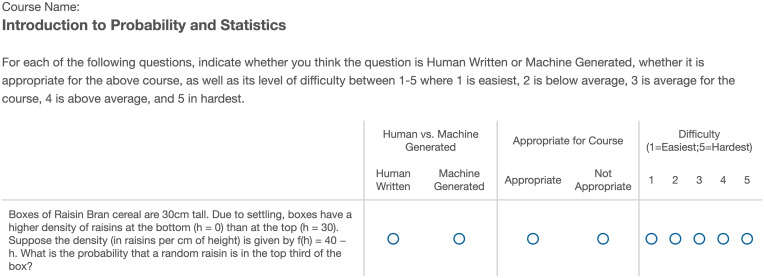
Student survey example question: For each of 60 questions, students are asked 1) if the question is human written or machine generated, 2) if the question is appropriate or inappropriate for the course, and 3) to rate the difficulty level of each question on a scale between 1 (easiest) and 5 (hardest).

For each of the 60 questions, the students are asked 3 survey questions: 1) “Is the question human-written or machine-generated?”, 2) “Is the question appropriate or not appropriate for the specific course?”, and 3) “What is the question’s difficulty level on a scale between 1 (easiest) and 5 (hardest)?”. An example of this survey format is given in Fig. 6. The students are asked to provide their ratings and not solve the questions. The survey is conducted online and anonymously.

## Results

### Questions Solved.

We solve 265 questions, 213 of them automatically, as described in *SI Appendix*. These 265 questions include 25 randomly sampled questions from each of the seven courses (18.01/18.02/18.03/18.05/18.06/6.042/COMS3251) and 15 randomly sampled questions for each of the six topics in the MATH dataset (Prealgebra/Algebra/Number Theory/Counting and Probability/Intermediate Algebra/Precalculus). The breakdown of automatic solve rate by zero-shot and few-shot learning using Codex compared with GPT-3 and GPT-3 with CoT is shown in Fig. 3. Programs involve step-by-step commands; therefore, CoT is inherent in programs.

### Visualization of Embedded Questions.

**Fig. 7.**
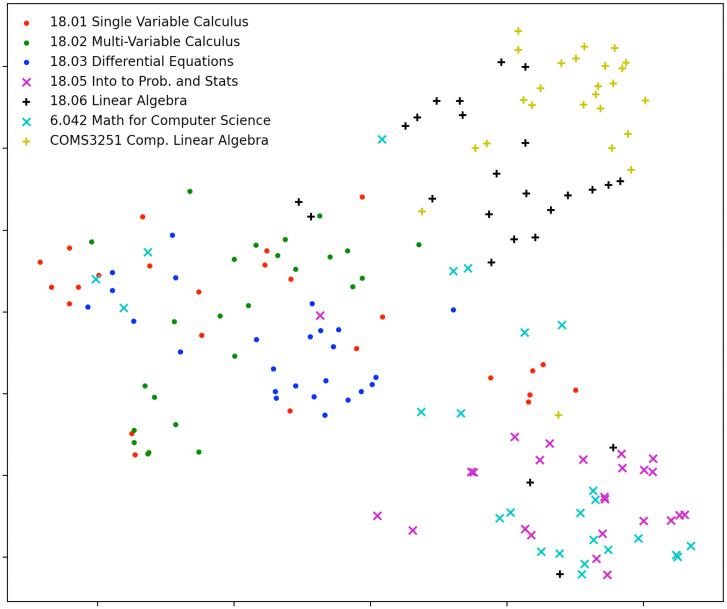
Visualization of embeddings of course questions: We embed the course questions into a 2,048-dimensional space using OpenAI’s text-similarity-babbage-001 embedding engine, which captures semantic similarity between texts. We then use uniform manifold approximation and projection to reduce the dimensionality to two. This shows distinctive clusters based on topics. We see clusters of questions from MIT’s 18.06 Linear Algebra and Columbia’s COMS3251 Computational Linear Algebra at *Top Right*. At *Left*, we see a cluster of the questions from MIT’s 18.01, 18.02, and 18.03. At *Bottom Right*, we see a cluster of the questions from MIT’s 18.05 Introduction to Probability and Statistics and 6.042 Mathematics for Computer Science, covering probability and statistics.

We embed the 175 mathematics course questions onto a 2,048-dimensional space using OpenAI’s text-similarity-babbage-001 embedding engine, which captures semantic similarity between texts. We then use uniform manifold approximation and projection (UMAP) (18,19) to reduce the dimensionality of the 175 question embeddings to 2. Fig. 7, the plot of these two dimensions, shows that the embedded questions are clustered by course topics. We see clusters of questions representing linear algebra from MIT’s 18.06 Linear Algebra and Columbia’s COMS3251 Computational Linear Algebra in Fig. 7, *Top Right*. In Fig. 7, *Left*, we see a collection of the questions representing calculus from MIT’s 18.01 Single Variable Calculus, 18.02 Multivariable Calculus, and 18.03 Differential Equations. In Fig. 7, *Bottom Right*, we see a cluster of the questions from MIT’s 18.05 Introduction to Probability and Statistics and 6.042 Mathematics for Computer Science, covering probability and statistics.

### Automatically Generating New Questions.

We generate new questions for each course and topic by prompting Codex with numbered human-written questions to generate the next question automatically. Specifically, we create prompts of 25 randomly selected problems for which Codex generates correct answers, remove the questions after a randomly chosen question in the list, and have Codex complete the next new question. We present 130 new questions generated by Codex in *SI Appendix* to demonstrate this capability. These include 10 new questions for each of the seven courses and each of the six MATH topics. Table 2 shows one generated question for each class and MATH topic. Generating a question takes less than 1 s. We can generate an arbitrarily large number of questions, demonstrating that this is a practical and effective method for creating new course content.

**Table 2.** Examples of new questions generated automatically by Codex for each course and the most similar question from its course

ID	Course	Machine-generated question	Most similar human-written question	Similarity
1	18.01 Single-Variable Calculus	Find the area of the region bounded by the curve and the *x* axis. *y* = *x*^2^ sin(*x*), 0 ≤ *x* ≤ π.	Find the area of the region under the given curve from 1 to 2. *y* = (*x*^2^ + 1)*/*(3*x* − *x*^2^).	0.61
2	18.02 Multi-Variable Calculus	Find *a* × *b*. *a* = ⟨9, −2, 1⟩, *b* = ⟨−2, 1, 1⟩	Find *a* × *b*. *a* = ⟨5, −1, −2⟩, *b* = ⟨−3, 2, 4⟩.	0.87
3	18.03 Differential Equations	Use the method of separable variables to solve the initial-value problem $\frac{dy}{dx}=5e^{x},y\left(2\right)=12$ when *x* = 2.	Separate variables and use partial fractions to solve the initial value problems. Use either the exact solution or a computer-generated slope field to sketch the graphs of several solutions of the given differential equation and highlight the indicated particular solution. *f*′(*x*) = 3*f* (*x*)(5 − *f* (*x*)), *f* (0) = 8.	0.21
4	18.05 Introduction to Probability and Statistics	Let *X* be a uniformly distributed random variable over the interval [0, 1). Find E[*X*^2^].	Let *X* be the result of rolling a fair four-sided die. Let *Y* be the result of rolling a fair six-sided die. You win 2*X* dollars if *X > Y* and lose 1 dollar otherwise. After playing this game 60 times, what is your expected total gain?	0.29
5	18.06 Linear Algebra	Write a Matlab code to determine whether the given matrix *A* = [1, 1; 4, 4] is positive semidefinite and if it is negative semidefinite.	Find *A*′*A* if the columns of *A* are unit vectors, all mutually perpendicular.	0.21
6	6.042 Mathematics for Computer Science	A student is taking a test consisting of *n* multiple-choice questions. Each question has five possible answers, and only one is correct. The student knows that the probability that any particular question is answered correctly is $\frac{1}{5}$ Let *X* be the number of questions answered correctly by the student. What is E(*X*)?	MIT students sometimes delay laundry for a few days. Assume all random values described below are mutually independent. A busy student must complete three problem sets before doing laundry. Each problem set requires 1 d with probability $\frac{2}{3}$ and 2 d with probability $\frac{1}{3}$ Let *B* be the number of days a busy student delays laundry. What is E(*B*)?	0.47
7	COMS3251 Computational	Find a combination of the vectors $\left[\begin{array}{ccc} 1 & 2 & 3\\ 4 & 5 & 6\\ 7 & 8 & 9 \end{array}\right]$ that gives the vector [123].	Find a combination of the vectors $\left[\begin{array}{ccc} 1 & 2 & 3\\ 4 & 5 & 6\\ 7 & 8 & 9 \end{array}\right]$ that gives the zero vector.	0.90
8	MATH Pre-Algebra	How many four-digit positive integers are there with hundreds digit 2?	How many four-digit positive integers are there with thousands digit 2?	0.90
9	MATH Algebra	Find the distance between the points (0, 0) and (3, 4).	Find the distance between the points (0, 4) and (3, 0).	0.99
10	MATH Number Theory	Find the smallest positive integer *n* such that *n*^2^ is divisible by 2^10^ and *n*^3^ is divisible by 3^10^.	How many four-digit numbers whose digits add up to 9 are divisible by 11?	0.25
11	MATH Counting and Probability	How many ways are there to divide a set of 10 objects into two sets of equal size?	Compute $\left(\begin{array}{l} 8\\ 4 \end{array}\right)$.	0.12
12	MATH Intermediate Algebra	Let *x* and *y* be positive real numbers such that *x*^2^ + *y*^2^ = 1. Find the maximum value of *xy*.	Given that *x*^2^ + *y*^2^ = 14*x* + 6*y* + 6, find the largest possible value of 3*x* + 4*y*.	0.59
13	MATH Precalculus	Let *A* be the matrix $\left[\begin{array}{ccc} 1 & 2 & 3\\ 4 & 5 & 6\\ 7 & 8 & 9 \end{array}\right]$. Find the determinant of *A*^2^ + *A*^3^.	If det(*A*) = 2 and det(*B*) = 12, then find det(*AB*).	0.41

### Student Survey Results.

**Fig. 8.**
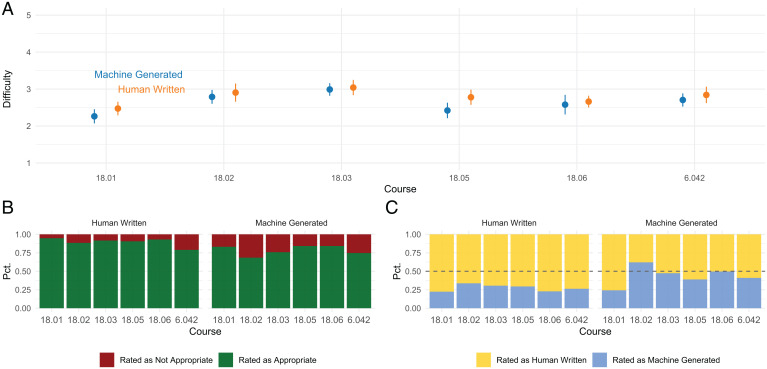
Student survey results. *A* compares the level of difficulty of human-written questions and questions generated by our approach for each course based on the student ratings. The plot shows the means of the difficulty ratings between 1 (easiest) and 5 (hardest) and their 95% CIs. *B* shows the percentage of human-written and machine-generated questions rated as appropriate and not appropriate for the course. *C* shows the percentage of human-written questions rated as human written or machine generated (*Left*) and the percentage of machine-generated questions rated as human written or machine generated (*Right*).

Fifteen participants completed our survey, answering questions about all 60 questions, taking a median of 40 min. Fig. 8 summarizes the results of the student survey comparing human-written and machine-generated questions. Fig. 8*A* compares the difficulty level of human-written questions and the machine-generated questions for each course based on the student ratings. The plot shows the means of the difficulty ratings between 1 (easiest) and 5 (hardest) and their 95% CI. Fig. 8*B* shows the percentage of human-written and machine-generated questions rated by students as appropriate or not appropriate for the courses. Fig. 8*C* shows the percentage of human-written questions rated as human written or machine generated (*Left*) and the percentage of machine-generated questions rated as human written or machine generated (*Right*).

The student survey results are summarized as follows:

• Survey participants rated our machine-generated and human-written questions to be similar in difficulty within CIs.
• Survey participants rated human-written questions slightly more appropriate for the courses than machine- generated ones.
• Survey participants rated human-written questions more likely to be human written as shown in Fig. 8 *C*, *Left*. Survey participants rated machine-generated questions equally likely to be machine generated and human written as shown in Fig. 8 *C*, *Right*.

### Human Level.

With our methodology, Codex reaches human performance levels in the contexts of both solving existing questions and generating new content. We achieve 81% automatic accuracy in solving mathematics course problems at MIT and Columbia, comparable to typical student performance on these problem sets in our MIT and Columbia University courses. Furthermore, we automatically generate new questions that are indistinguishable to students from human-written course questions.

### Implementation Details.

We make our data and code publicly available (19). We use the latest version of OpenAI’s GPT-3 text-davinci-002 and Codex codex-davinci-002 engines for all of our experiments. We fix all of Codex’s hyperparameters to be the same for all solution and explanation experiments to yield deterministic and reproducible results. Specifically, top P, which controls diversity, is set to 0 and sampling temperature, which controls randomness, is also set to 0. The frequency and presence penalties are set to 0, and we do not halt on any stop sequences. We allow diversity and randomness for all new question generation experiments by setting the top P and temperature to 0.1. Each prompt is structured as a Python documentation comment surrounded by triple quotations and line breaks. We evaluate the solution by running the generated program using a Python interpreter. Evaluations are considered correct if the printed output or the value returned by the generated program is the correct solution.

Few-shot learning prompts are structured as follows: For each question–code example being used, we insert the question in a docstring on the following available line, have a line break, and then insert the code on the following lines. After all the examples, we insert the target question at the end in the same way as described above and prompt Codex.

CoT prompts for GPT-3 are implemented by adding the text “Let’s think step by step” (7) after the few-shot questions and answers and the new question.

### Types of Problems the Model Cannot Solve.

There are a few different types of problems the model is incapable of solving: 1) any problem for which the question is in the form of an image or other nontext modality; 2) questions with solutions that require proofs; and 3) problems that are computationally intractable, such as factoring very large primes. This last category is not expected in any math course assignment, as students themselves would also be unable to answer them. That being said, many questions that students can answer have generalizations that are computationally intractable.

## Conclusion

We demonstrate that few-shot learning and program synthesis using OpenAI Codex is able to solve, explain, and generate university-level mathematics problems at a human level. In contrast, previous methods using transformers pretrained only on text, such as GPT-3, fail on these tasks. We verify that our strong results are not overfitting the training data by solving a new course that is not available online. We also generate and analyze new problem sets. The success of this work confirms that programs serve as a good representation and computation environment for solving math problems. Since our approach requires no additional training, it is easily scalable. This work addresses significant pedagogical challenges, bringing substantial benefits to higher education like curriculum design and analysis tools and automatic content generation.

We show that neural network synthesis with modern programming languages is more dynamic and widely applicable than expression trees and likely solves a broader range of problems. Although any finite computation could be expressed as a sufficiently large expression tree, one may see an arbitrarily large expansion in the size of the expression tree needed, as opposed to a Turing-complete language. This flexibility is bolstered by the massive corpus of existing programs, which eclipses the number of labeled expression trees available. Program outputs are also inherently more human readable, as the ability to use abstraction, modularity, and high-level logic leads to more explicit illustrations of the path to a solution. Furthermore, program synthesis can convey logical deductions directly through explanatory comments and function and variable names. In particular, we see such descriptive text and derivations in a number of the Codex outputs. The unification of such formal and informal language is an inherent advantage of our methodology. We emphasize that the results may be complex and multimodal. For example, by using packages such as Matplotlib, we can produce graphs of equations. This advanced and unique ability is time consuming for humans and offers a significant pedagogical benefit.

In summary, we automatically solve, explain, and generate university-level mathematics course questions in real time at a human level (20). Students rated machine-generated questions as equally likely to have been human written as machine generated. Students also rated machine-generated questions as similarly difficult to human-written questions and most appropriate for their respective courses. Finally, we have succeeded in scaling up this work to over 30 STEM courses across 13 departments in science and engineering schools at MIT and Ivy League universities, with excellent results.

## Supplementary Material

Supplementary File

## Data Availability

Data and code have been deposited in Github (https://github.com/idrori/mathQ).

## Footnotes

^*^American Mathematics Competitions.

^†^American Invitational Mathematics Examination.
